# Supplementary material for: Exploring the Language Used to Describe Older Patients at Multidisciplinary Cancer Conferences
Source: Cancers (Basel). 2024 Apr 12;16(8):1477. doi: 10.3390/cancers16081477 (PMC11047842; doi:10.3390/cancers16081477)
Supplement: Supplementary file 1 [file cancers-16-01477-s001.zip › cancers-2917252-supplementary.pdf]

Supplementary Table S1: Presentation of frailty-related descriptors and GA domains by MCC site

Variable	Site A	Site B	Site C	Site D	Chi-square *p*-value
**Frailty or Fitness, %**	26.7	11.1	26.3	15.8	0.616
**Age, %**	53.3	11.1	36.8	36.8	0.072
**Overall health, %**	13.3	11.1	15.8	42.1	0.112
**Performance status, %**	6.7	11.1	0	26.3	0.069
**Treatment tolerance, %**	40	22.2	15.8	47.4	0.139
**GA domains, ≥ 2 mentioned, %**	13.3	83.3	5.3	21.1	**<0.001**

Note: "GA" = Geriatric Assessment, "MCC" = Multidisciplinary Cancer Conference, "GI" = Gastrointestinal, "GU" = Genitourinary.
Note: Fisher-Freeman-Halton Exact Test was used instead of Pearson Chi-Square, where applicable, due to low counts.

Supplementary Table S2: **Presentation of frailty-related descriptors and GA domains by presenter specialty**

Variable	Medical Oncology (n=22)	Radiation Oncology (n=7)	Surgical Oncology (n = 44)	Chi-square *p*-value
**Frailty or Fitness, n (%)**	5 (22.7)	2 (28.6)	7 (15.9)	0.605
**Age, n (%)**	8 (36.4)	3 (42.9)	15 (34.1)	0.935
**Overall health, n (%)**	3 (13.6)	2 (28.6)	11 (25.0)	0.569
**Performance status, n (%)**	6 (27.2)	0 (0)	2 (4.5)	**0.019**
**Treatment tolerance, n (%)**	5 (22.7)	4 (57.1)	15 (34.1)	0.246
**GA domains, ≥ 2 mentioned, n (%)**	5 (22.7)	0 (0)	17 (38.6)	0.085

Note: "GA" = Geriatric Assessment.
Note: Fisher-Freeman-Halton Exact Test was used instead of Pearson Chi-Square, where applicable, due to low counts.

Supplementary Table S3: **Presentation of frailty-related descriptors and GA domains by presenter training level**

**Variable**	**Trainee (n=33)**	**Faculty (n=40)**	**Chi-square *p*-value**
**Frailty or Fitness, n (%)**	7 (21.2)	7 (17.5)	0.770
**Age, n (%)**	6 (18.2)	18 (45.0)	**0.024**
**Overall health, n (%)**	7 (21.2)	9 (22.5)	1.000
**Performance status, n (%)**	7 (21.2)	1 (2.5)	**0.019**
**Treatment tolerance, n (%)**	9 (27.3)	12 (30.0)	1.000
**GA domains, ≥ 2 mentioned, n (%)**	18 (54.5)	6 (15.0)	**<0.001**

Note: "GA" = Geriatric Assessment.
Note: Fisher's Exact Test was used instead of Pearson Chi-Square, where applicable, due to low counts.
